# Ophthalmic nurses: vital team members in the push for better eye health

**Published:** 2020-12-31

**Authors:** Stella Antwi-Boasiako, Heather Machin

**Affiliations:** 1 Principal: Ophthalmic Nursing School, Korle-Bu, Ghana.; 2 Consultant: Fred Hollows Foundation NZ and Project Officer: Centre for Eye Research Australia, University of Melbourne, Australia.

**Ophthalmic nurses improve local and global eye health and contribute to people-centred eye care.**

Ophthalmic nurses are qualified nurses who have received additional training in eye care. They can provide advice during eye health emergencies and provide vision screening, refraction, and optical dispensing. Ophthalmic nurses also play a central role in the planning and delivery of people-centred eye care services, including the delivery of comprehensive eye care programmes, systems and policy development, and integration with other health specialties. This supports the collective goal of ensuring universal health coverage - including eye health - for all. The World Health Organization (WHO) defines universal health coverage as: “… ensuring that all people have access to the promotive, preventive, curative and rehabilitative health services they need, of sufficient quality to be effective, while also ensuring that people do not suffer financial hardship when paying for these services.”

**Landmark year for nursing**

2020 was the World Health Organization (WHO) Year of the Nurse and Midwife; it was also the 200th anniversary of the birth of Florence Nightingale, who is widely revered as the founder of modern nursing.

Nurses work across different communities and settings, both urban and rural, and can therefore offer patient-centred care close to where people live. We therefore call on eye care leaders worldwide to recognise the vital role of ophthalmic nurses and other allied health personnel in supporting the push for universal health coverage.

There are many areas in which nurses can contribute to people-centred eye care and improve local and global eye health. We propose that policy makers, programme managers, hospital managers, nurses, other allied health personnel, and the rest of the eye team work together to implement the following suggestions.

## Leadership and advocacy

- Promote people-centred care. Work with policy makers and decision makers to ensure that patients can move across the whole patient pathway: from the community, to secondary and tertiary services, to low vision and rehabilitation services (where nurses are also involved). Integrate eye health into other health areas and improve referral between services.
- Recognise ophthalmic nursing in the human resources framework, set their payscale to match that of other nursing specialties, and include nurses in leadership teams.

## Prevention and health promotion

- Participate in the development and implementation of education and community awareness campaigns.
- Educate the public, e.g., on radio and television talk shows or at local community centres (provided this is allowed by current social distancing guidelines to protect against COVID-19.
- Participate in national and global awareness campaigns.
- Ensure equity in access to eye health by training community health nurses and volunteers to identify and manage simple eye conditions in the community. The WHO Africa *Primary Eye Care Training Manual* is a useful resource (**www.afro.who.int/publications/primary-eye-care-training-manual**).
- Organise eye screening in schools, markets, organisations, and churches, even at various organisations' annual general meetings, if COVID-19 guidelines permit this.
- Develop educational materials and tools to raise awareness about eye health.

## Direct patient care

- Put patients at the centre of the care nurses provide - consider their needs and experiences, e.g., by developing and implementing patient focus groups and providing opportunities for patient feedback (see ‘Putting patients at the centre of eye care’, **bit.ly/CEHJ78**). Good communication with patients and their family members or carers is vital.
- Focus on safety and risk prevention. For example, implement the safe site surgical checklist (WHO, 2009).^2^
- Explore skills exchange (also known as task sharing/task shifting). This may include learning new skills that may be traditionally considered another professional's job. For example, ophthalmic nurses and other allied health personnel can support ophthalmologists in screening, preoperative and postoperative care of cataract, and surgical assisting; this ultimately contributes to improved output, productivity, and outcomes. The International Council of Ophthalmology has launched an initiative^2^ to examine skills exchange through a formal, planned framework and training programme. Non-nurses can also be taught to carry out tasks that may traditionally be considered nursing tasks.

**WHO key facts on nurses and midwives^1^**

1. Globally, nurses are the largest group of health care providers, representing almost 50% of the health workforce at all levels.^1^
2. There is a global shortage of health workers. Nurses and midwives are the group with the greatest shortages - together they represent more than half of the global shortage in health workers. The problem is greatest in South East Asia and Africa.
3. For all countries to reach the United Nations Sustainable Development Goal 3 on health and wellbeing, WHO estimates that the world will need an additional 9 million nurses and midwives by the year 2030.
4. Nurses play a critical role in health promotion, disease prevention and the delivery of primary and community care. They provide care in emergency settings and will be key to the achievement of Universal Health Coverage.
5. Achieving health for all will depend on there being sufficient numbers of well-trained and educated, regulated, and well-supported nurses and midwives, who receive pay and recognition commensurate with the services and quality of care that they provide.
6. Investing in nurses and midwives is good value for money. The report of the UN High Level Commission on Health Employment and Economic Growth concluded that investment in education and job creation in the health and social sectors result in a triple return of improved health outcomes, global health security, and inclusive economic growth.
7. Globally, 70% of the health and social workforce are women, compared to 41% in all employment sectors. Nursing and midwifery occupations represent a significant share of the female workforce.

**From the editor**

The roles and responsibilities of eye care personnel, and the terminology used to describe them, vary significantly from one country to another. The responsibilities described in this article, and in this issue of the *Community Eye Health Journal* may also fit the role descriptions of clinical officers, ophthalmic clinical officers, ophthalmic assistants, or other roles included in the term ‘allied ophthalmic personnel.’

## Quality improvement

- Foster a continuous quality improvement approach to service provision; for example, by welcoming, conducting, and acting on internal or external audits and patient feedback surveys, as well as your own professional development.
- Keep up to date with your own professional development, and new developments in the field, by:
  – Updating your qualifications
  – Taking part in professional development activities
  – Keeping abreast of what is happening in the care sector, and/or an area of specialty that is directly related to your work, by participating in local continuing professional development opportunities
  – Reading journals and articles (such as in the *Community Eye Health Journal*)
  – Taking part in webinars (see **www.iapb.org**), or by taking an online course. There are many free or low-cost courses available online, including from the International Centre for Eye Health (**iceh.lshtm.ac.uk/oer/**), Cybersight (**cybersight.org/online-learning/**), WHO (**openwho.org/courses**) and JCAHPO (**eyecarece.jcahpo.org**).
- Take responsibility for the conditions in your workplace; for example, by reporting malfunctions and hazards.
- Take responsibility for the resources in your workplace. Put the safe storage and use of equipment, instruments, and consumables at the forefront of your daily practice.
- Contribute to the Global Green and Healthy Hospitals scheme (**www.greenhospitals.net**); for example, by considering how your hospital can reduce the amount of waste it produces or by proposing an action plan to reduce your carbon emissions.

## Building the eye care team

- Join, or build, a national or regional ophthalmic nursing special interest group/association.
- Welcome and encourage people into nursing, and then into the eye care subspecialty. Many countries need more ophthalmic nurses, so every nurse has a duty to promote the profession and welcome newcomers.
- Advocate for ophthalmic nurses to be included in the human resources framework and pay scales.
- Train nurses in departments such as endocrinology/diabetes care, or in geriatric care (care for older people).
- Foster collaborative, open and proactive relationships among all members of the eye care and/or hospital team. Team members may include ophthalmologists, optometrists, orthoptists, technicians, pathologists, and so on. The more we communicate, and the more we break down outdated and unhelpful barriers, the greater the likelihood that we will achieve the goal of universal health care for all.
- Help to grow the body of evidence needed to assist in the prevention and treatment of vision impairment and blindness by conducting research and by presenting and sharing your knowledge.
